# Biliary atresia: insights into mechanisms using a toxic model of the disease including Wnt and Hippo signaling pathways and microtubules

**DOI:** 10.1038/s41390-024-03335-9

**Published:** 2024-06-25

**Authors:** Sophia Fried, Adi Har-Zahav, Yara Hamudi, Sarah Mahameed, Rasha Mansur, Miri Dotan, Tal Cozacov, Raanan Shamir, Rebecca G. Wells, Orith Waisbourd-Zinman

**Affiliations:** 1 Institute for Gastroenterology, Nutrition and Liver Diseases, Schneider Children’s Medical Center of Israel, Petach Tikva, Israel; 2 Faculty of Medicine and Health Sciences, Felsenstein Medical Research Center, Tel-Aviv University, Tel-Aviv, Israel; 3 Division of Gastroenterology and Hepatology, Department of Medicine, Perelman School of Medicine at the University of Pennsylvania, Philadelphia, PA USA; 4 Department of Bioengineering, University of Pennsylvania, Philadelphia, PA USA; 5 Division of Gastroenterology, Hepatology, and Nutrition, Department of Pediatrics, The Children’s Hospital of Philadelphia, Philadelphia, PA USA

## Abstract

**Background:**

Mechanisms underlying bile duct injury in biliary atresia (BA) remain unclear and mechanisms of bile duct repair are unknown. This study aimed to explore the roles of microtubule instability and Wnt and Hippo signaling pathways in a biliatresone-induced BA model.

**Methods:**

Using primary murine neonatal cholangiocytes in both 2D and 3D cultures, and ex-vivo extra hepatic bile ducts (EHBD) which also has peri-cholangiocyte area, we analyzed injury and recovery processes. Injury was induced by the toxin biliatresone and recovery was induced by toxin wash-out.

**Results:**

Microtubule stabilizer paclitaxel prevented biliatresone-induced injury, both to cholangiocytes as well as reduced periductal αSMA stain, this process is mediated by decreased glutathione levels. RhoU and Wnt11 (Wnt signaling) and Pard6g and Amotl1 (Hippo signaling) are involved in both injury and recovery processes, with the latter acting upstream to Wnt signaling.

**Conclusions:**

Early stages of biliatresone-induced EHBD injury in cholangiocytes and periductal structures are reversible. Wnt and Hippo signaling pathways play crucial roles in injury and recovery, providing insights into BA injury mechanisms and potential recovery avenues.

**Impact:**

- Microtubule stabilization prevents cholangiocyte injury and lumen obstruction in a toxic model of biliary atresia (biliatresone induced).
- Early stages of biliatresone-induced injury, affecting both cholangiocytes and periductal structures, are reversible. Both Wnt and Hippo signaling pathways play a crucial role in bile duct injury and recovery, with a noted interplay between the two.
- Understanding mechanisms of cholangiocyte recovery is imperative to unveil potential therapeutic avenues.

## Introduction

Biliary atresia (BA) is a neonatal pan-cholangiopathy marked by the obliteration of the extrahepatic biliary system, leading to bile flow obstruction and subsequent liver fibrosis. Despite being the predominant reason for pediatric liver transplants, the etiology and pathogenesis of BA remain elusive^1^. Thus understanding the mechanisms of bile duct injury is of great importance.

Previous studies showed that the plant toxin biliatresone causes BA-like disease in zebrafish and murine cholangiocytes and it acts by decreased cellular glutathione (GSH)^2,3^. We used biliatresone as a valuable model to better understand mechanisms of cholangiocyte injury and to explore the pathways involved in the bile duct injury and recovery in a toxic model; Utilizing biliatresone, we previously studied mechanisms of bile duct injury and associated decreased GSH with Wnt signaling pathway members - RhoU, Hey2, and Sox17^2,4^. Furthermore, biliatresone-treated cholangiocytes exhibited reduced α-tubulin staining and consequent loss of cell-cell adhesion and increased epithelial permeability^2^. Despite observing a decline in microtubules expression, their exact role in injury remains under-investigated and is one of the scopes of the current study. Microtubules are polymers of tubulin that constitute a major part of the cytoskeleton and are known as regulators of directional cell migration, vesicle and organelle trafficking and mitosis, and play an important role in lumen formation and the maintenance of polarity in epithelial cells^5,6^. Interestingly, polarity abnormalities have been reported in human BA; a recent study found that extracellular matrix proteins and adhesion molecules that mediate cellular polarity and integrity are abnormal in BA patients^7^.

While there have been no reports of patients recovering from biliary atresia, understanding the underlying mechanisms of bile duct recovery could provide insights into potential future therapeutics. We showed in vitro that cholangiocyte organoids occluded in response to biliatresone may re-form their lumens after toxin washout^2^, however mechanisms of recovery of cholangiocytes are unknown.

This study aims to elucidate the role of microtubules in biliatresone-induced injuries and assess if paclitaxel, a microtubule stabilizer^8^, leads to decreased injury. We also seek to define the molecular mechanisms underpinning bile duct recovery. Given the established roles of the Wnt pathway in liver development^9,10^ and the Hippo signaling pathway in cell self-renewal and expansion^11^, we hypothesize their significance in the recovery process.

## Methods

### Cell culture

Primary neonatal (3 days old) extrahepatic cholangiocytes were used for 2D or 3D spheroid culture as previously described^2^. Small intrahepatic cholangiocyte cell line (SBEC) were used only for transfections with overexpression plasmids. Cells were cultured with biliary epithelial cell (BEC) medium and incubated at 37 °C with 5% CO_2_^2^.

### Spheroid culture

Primary extrahepatic cholangiocytes were cultured in 3D in a collagen-Matrigel mixture as described previously^12^. Cholangiocytes in 3D culture replicate, polarize, and form hollow spheroids with apical markers on the luminal side and basolateral markers on the external side after 7-8 days. Spheroids were used for experiments at day 8 after plating^12^.

### Neonatal bile duct explant culture

Intact EHBD were isolated from 0- to 3-day-old or 3-week-old BALB/c mice, cultured in a Vitron Dynamic Organ Culture Incubator, and treated with biliatresone or vehicle for 1, 4, 6, 24 or 30 h as described in detail in our previous study^4^. Bile ducts were then processed to paraffin blocks and sectioned for staining (see below, fluorescent staining).

### Biliatresone treatments

Biliatresone was synthesized as described^13^. Cells or tissues were treated with vehicle (DMSO), biliatresone (3 µg/ml), buthionine sulfoximine (BSO) (100 µM, Merck, catalog #19176), paclitaxel (10 µM 1:500, Tocris, catalog #1097), or nocodazole (10 µM 1:500, Tocris, catalog #1228) for 6 h unless otherwise noted.

### Transfection

Transfections of SBEC, primary cholangiocytes, or spheroid cultures were performed with Lipofectamine 2000 for plasmids (Invitrogen, catalog #11668019) and Dharmafect1 for siRNA (Dharmacon, catalog #T-2001-02) following the manufacturers’ instructions. The Plasmid was Wnt11 (pcDNA-Wnt11-V5 Addgene). Cells transfected with an empty plasmid with the same backbone served as controls for transfection experiments. For siRNA experiments, the Dharmacon ON-Target plus mouse siRNAs for Nf2, Pard6G and Amotl1 (GAAUGAAAUCCGAAACAUC, ACUACAAGUCACCGUGGAA, GCCAAUAGGUACUCUGUAA respectively) were used. Cells transfected with non-targeting siRNA served as controls for these experiments.

### Immunofluorescence staining

#### Spheroid culture

Treated spheroids were fixed with 4% paraformaldehyde (PFA), permeabilized with permeabilization solution (10% FBS, 0.5% Triton X-100 in PBS), and stained for various fluorescent markers: F-actin (1:1000; phalloidin tetramethyl rhodamine B isothiocyanate; Santa Cruz Biotechnology, catalog #301530) and α-tubulin (T9026 Sigma-Aldrich) or keratin19 (K19, Developmental Studies Hybridoma Bank, TROMAIII) or ZO-1 (Abcam ab96587). Spheroids were immunofluorescently stained as previously described^4^.

Images were obtained using a Zeiss Lsm800 confocal microscope with 40X magnification. Images were taken at the level of the midsection of each spheroid, where the luminal diameter was greatest.

#### Cholangiocyte 2D culture

SBECs (0.5 × 10^5^ cells per well (0.7 cm^2^)) were grown in 8-well chamber slides (LAB-TEK II Nunc, Thermo Fisher Scientific). Cells were treated with biliatresone, BSO or DMSO for 24 h and fixed with 4% PFA (30 min 37 °C). In transfection experiments with plasmids for overexpression, cells were fixed after 48 h in the same manner. We then proceeded to immunofluorescent staining as described in the previous paragraph and detailed in our previous study^4^. Cells were visualized using an Axioimager Z2 Apotome microscope, at 40X. Images were analyzed using ImageJ software, Win64 version: https://imagej.net/Fiji/Downloads.

#### Staining of paraffin-embedded sections

Slides with paraffin-embedded tissue sections were stained for various antibodies^4^. Briefly, slides were deparaffinized by warming to 60 °C, treated with xylene, then rehydrated with decreasing concentrations of ethanol (100%, 95%, 80%, 70%). Slides were incubated in microwave oven for 15 min in citric acid buffer (pH 6), cooled, and washed in running water, washed with PBS, blocked with PBT, and cultured with primary antibodies diluted in PBT over-night at 4 °C, washed with PBT, incubated with secondary antibodies for 30 min in RT, washed two times, stained with DAPI (5 min, RT), washed, and covered with mounting medium and a cover slip. Images were taken using an Axioimager Z2 Apotome microscope at 20X-40X magnification.

#### Whole mount staining

Neonatal mouse (1 week old except if otherwise specified) EHBDs were stained using a whole‐mount staining technique as previously described^2^.

EHBD were stained with the following antibodies: cholangiocyte marker K19 (1:10, Developmental Studies Hybridoma Bank, TROMAIII), myofibroblast marker α-smooth muscle actin (1:100 Abcam ab7817) vimentin (1:250 Abcam AB92547). Images were taken using a Leica SP5 confocal microscope at 40X magnification. Images were analyzed by using FIJI ImageJ software, Win64 version: https://imagej.net/Fiji/Downloads.

### Rhodamine efflux assay

Assay of rhodamine efflux from spheroids, a measure of monolayer permeability, was modified from a published protocol^2^. On day 8, spheroids were incubated with rhodamine 123 (100 µM, Sigma) and CellMask Orange (C10045, Thermo Fischer) for 15 min followed by five washes with phenol-free DMEM (Gibco, 21041025) and were then treated with either biliatresone or DMSO or a combination of biliatresone with paclitaxel; live cells were imaged every 20 min for 2 h after treatment using a Zeiss Lsm800 confocal microscope. The average percent of rhodamine reduction was quantified by calculating the difference between the mean fluorescence intensity at t = 0 and t = 2 h for each set of images by using FIJI ImageJ software, Win64 version: https://imagej.net/Fiji/Downloads. Next, we calculated the average percent of mean fluorescent intensity reduction for each condition of the experiment (DMSO, biliatresone and biliatresone+paclitaxel).

### Real-time quantitative PCR (qRT-PCR)

Primary cholangiocytes or SBECs (3 × 10^5^ cells in 2 ml per well in 6 well plates) were plated at 37 °C to allow cell adhesion. Cells were treated with either biliatresone/DMSO for 6 h or biliatresone for 6 h followed by a 24 h washout. Total RNA from the cells was extracted with the EZ-10 DNAaway RNA Mini-Preps Kit (Bio Basic, catalog # BS88133) according to the manufacturer’s protocol. cDNA was prepared using a qScript TM cDNA Synthesis kit (Quantabio, Beverly, MA). Primer sets for:

RhoU (TGTCTGTAGATGGGCGGCCTGT, TTCTGGAAGGATGTGGGGCTCA), Nf2 (ATAAAAAGGGCACAGAGTTG, AATAGTAAACTCCTTGTCGC), Amotl1 (AAAGTTGGAAATGGAGTTGG, CTTCTCTCGTAACTCTTCCTC),

Wnt11 (CCAATAAACTGATGCGTCTAC, ATTTACACTTCGTTTCCAGG),

Pard6G (CTGTGAATGATGAAGTCCTG, GTTGGCTATCATCATGTCTG) and Rp13a (AGGGGCAGGTTCTGGTATTG, TGTTGATGCCTTCACAGCGT) were used. Rp13a was our endogenous control. Real-time quantitative PCR was performed using the StepOnePlus real-time PCR system. Expression analysis was done using the double delta ct method as previously described^14^.

### PCR array profiling

#### RNA purification

Primary cholangiocytes were treated with: biliatresone for 6 h, DMSO for 6 h, or biliatresone for 6 h followed by 24 h of washout with fresh media. RNA was purified using the miRNeasy® Mini Kit (Qiagen, catalog #217004) according to the manufacturer’s protocol including the on-column DNase digestion step.

#### cDNA synthesis and amplification

1.5 μg of total RNA from each sample was reverse transcribed using the first-strand cDNA synthesis kit (Qiagen, catalog #330401), including the genomic DNA elimination step (5 mins, 42 °C). Following the manufacturer’s protocol, the mix was then incubated at 42 °C for 15 min, then at 95 °C for 5 min.

#### PCR array qRT-PCR

The amplified cDNA was then diluted with nuclease-free water and added to the RT 2 qPCR SYBR Green Master Mix (Qiagen, catalog #330501). 25 μl of the experimental cocktail was added to each well of the mouse Wnt Signaling Pathway PCR array (Qiagen, PAMM-043Z, catalog #330231) or the mouse Hippo Signaling Pathway PCR array (Qiagen, PAMM-172ZC, catalog #330231). Real-time PCR was performed and all steps were done according to the manufacturer’s protocol for the ABI StepOne Plus. Ct values were then deposited on the Geneglobe website for analysis. All samples passed the quality tests for PCR Array reproducibility, reverse transcription efficiency, and genomic DNA contamination. Genes were normalized using three different housekeeping genes (Hsp90ab1, B2m, and Gusb).

### KEGG pathway scheme generation

We used the KEGG (Kyoto Encyclopedia of Genes and Genomes) application programming interface in order to generate the functional maps of Hippo and Wnt pathways. KEGG is widely used as a reference database of pathway networks for integration and interpretation of large-scale datasets generated by high-throughput sequencing technology and provides useful information for predicting functional profiles of genes.

### Statistical and image analysis

Values are presented as mean ± Standard errors unless otherwise indicated; a two-tailed student t-test was used for comparison. A significant difference between groups was defined as *p* < 0.05. All experiments were conducted at least 3 times with duplicates (number specified at each experiment).

Images were analyzed by using FIJI ImageJ software, Win64 version: https://imagej.net/Fiji/Downloads. A standard color threshold was applied.

α-tubulin relative intensity was calculated. Quantification of total α-SMA positive area in the bile ducts was automated with a custom ImageJ macro (MeasureSignalWidthV5.ijm) with values that were normalized to control.

Vimentin was compared between samples by measuring the average thickness of vimentin staining surrounding the lumen (*n* = 20 ducts).

### Study approval

All mice used in experiments were under a strict standard of care and experimental planning, covered by licensed approval from the Tel Aviv University Institutional Review Board and the Israeli Ministry of Health (License number 01-16-098). BALB/c mice were obtained from ENVIGO Israel. Both male and female mice were used for all analyses.

## Results

### Biliatresone-induced microtubule instability is mediated by decreased GSH and prevented with paclitaxel

**Fig. 1:**
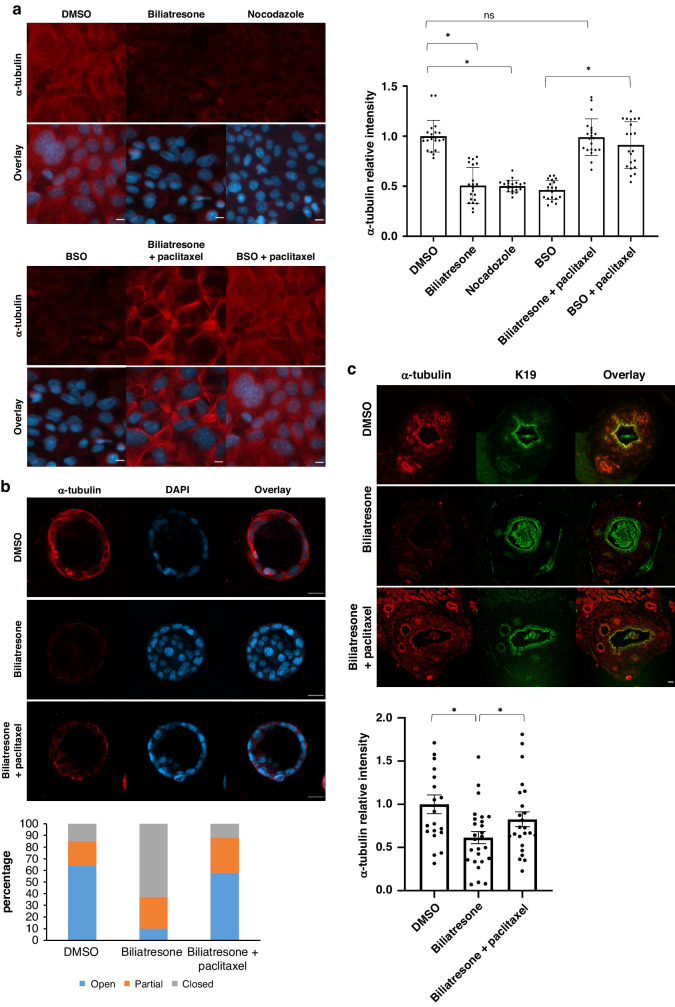
Paclitaxel prevents biliatresone-induced microtubule injury. **a** Primary neonatal mouse cholangiocytes treated with DMSO, biliatresone, nocodazole, BSO, biliatresone with paclitaxel or BSO+paclitaxel for 6 h and immunostained for DAPI (blue) and α-tubulin (red), *n* = 3 (20 images of each condition were quantified); α-tubulin staining quantification (relative mean fluorescence intensity) shown in the graph below. Standard errors reflected as error bars, (*) represents *p* < 0.01 (DMSO vs biliatresone *p* < 0.00001, DMSO vs. biliatresone +paclitaxel *p* = 0.85, biliatresone vs. biliatresone+paclitaxel *p* < 0.00001, DMSO vs. nocodazole *p* < 0.00001, BSO vs. BSO+paclitaxel *p* < 0.00001). Scale bars: 20 µm. **b** Primary neonatal mouse cholangiocytes grown in 3D culture as spheroids treated with the above treatments and stained for DAPI (blue), α-tubulin (red) and K19 (green). Spheroids were classified as open, partially open or closed, and quantified for each condition, *n* = 3 (67, 89 and 50 spheroids were quantified for DMSO, biliatresone and biliatresone+paclitaxel treatments respectively). Scale bars: 50 µm. **c** Neonatal mouse EHBD explants were cultured in a high-oxygen environment with the above treatments. EHBD were immunostained for K19 (green) and α-tubulin (red) and quantified, *n* = 3 (20,26 and 25 images were quantified for DMSO, biliatresone and biliatresone+paclitaxel treatments respectively), Error bars refer to standard error. (*) represents *p* < 0.05 (*p* = 0.0009, *p* = 0.03). Scale bars: 20 µm.

Biliatresone treatment has been shown to decrease cellular α-tubulin staining^2^. In order to determine the role of microtubule instability in biliatresone-induced injury processes we treated cholangiocytes with the microtubule stabilizing agent paclitaxel. Cholangiocytes in culture treated with paclitaxel along with biliatresone (biliatresone+paclitaxel group) maintained the typical α-tubulin staining of non-biliatresone-treated cells (DMSO group). Quantification of α-tubulin staining showed a 50% reduction in biliatresone-treated cholangiocytes (biliatresone group) while with biliatresone plus paclitaxel group, α-tubulin staining was not significantly different from control (DMSO group) (*p* < 0.001 and *p* = 0.85 respectively) (Fig. 1a). Next, we applied nocodazole, which destabilizes microtubules by blocking the polymerization of tubulin into microtubules. Cholangiocytes in culture treated with nocodazole had significantly decreased α-tubulin staining, phenotypically mimicking biliatresone effect (Fig. 1a). Biliatresone binds and decreases intracellular GSH^15,16^. Buthionine sulfoximine (BSO), which reduces GSH, phenocopies biliatresone effect on cholangiocyte spheroids. Microtubule disruption due to decreased GSH was described in various cell types^17,18^. We thus wanted to determine if paclitaxel would also prevent BSO-induced injury.

While α-tubulin stain decreased by 54% in cholangiocytes treated with BSO (*p* < 0.000001), the addition of paclitaxel prevented this decrease (compared to DMSO *p* = 0.16) (Fig. 1a).

Next, we wanted to determine the effect of microtubule stabilization on cholangiocytes grown in 3D spheroids. While biliatresone treatment of cholangiocyte spheroids resulted in lumen obstruction, the addition of paclitaxel left the spheroid structure intact. We graded the spheroids in each treatment condition as open (lumen wide open), partially closed (small lumen), or closed (obstructed lumen) (Fig. 1b). Biliatresone treatment resulted in lumen obstruction in the majority of the spheroids with only 10.11% open lumens while the addition of paclitaxel to biliatresone resulted in 58% open spheroids (*p* = 0.00001).

Next we wanted to assess whether microtubule stabilization prevents biliatresone-induced effects an ex vivo extra-hepatic bile duct model, as that allows assessment of cholangiocytes in their original shape with the addition of the peri-ductal surrounding. We treated neonatal EHBD explants in a high oxygenation rotating incubator with biliatresone with and without paclitaxel. EHBD treated with biliatresone demonstrated disruption of the cholangiocyte monolayer and lumen obstruction (Supplementary Fig. 1a), and reduced α-tubulin staining compared with DMSO-treated ducts (Fig. 1c). Both lumen obstruction and microtubule disruption were prevented with the addition of paclitaxel (Fig. 1c and Supplementary Fig. 1a). The addition of paclitaxel to BSO treatment also resulted in prevention of BSO-induced lumen obstruction (Supplementary Fig. 1a). Altogether, microtubule stabilization prevents cholangiocyte injury and lumen obstruction caused by biliatresone or decreased GSH.

### Microtubule stabilization prevents biliatresone-induced increased permeability, tight junction disruption and increased periductal α-SMA expression

**Fig. 2:**
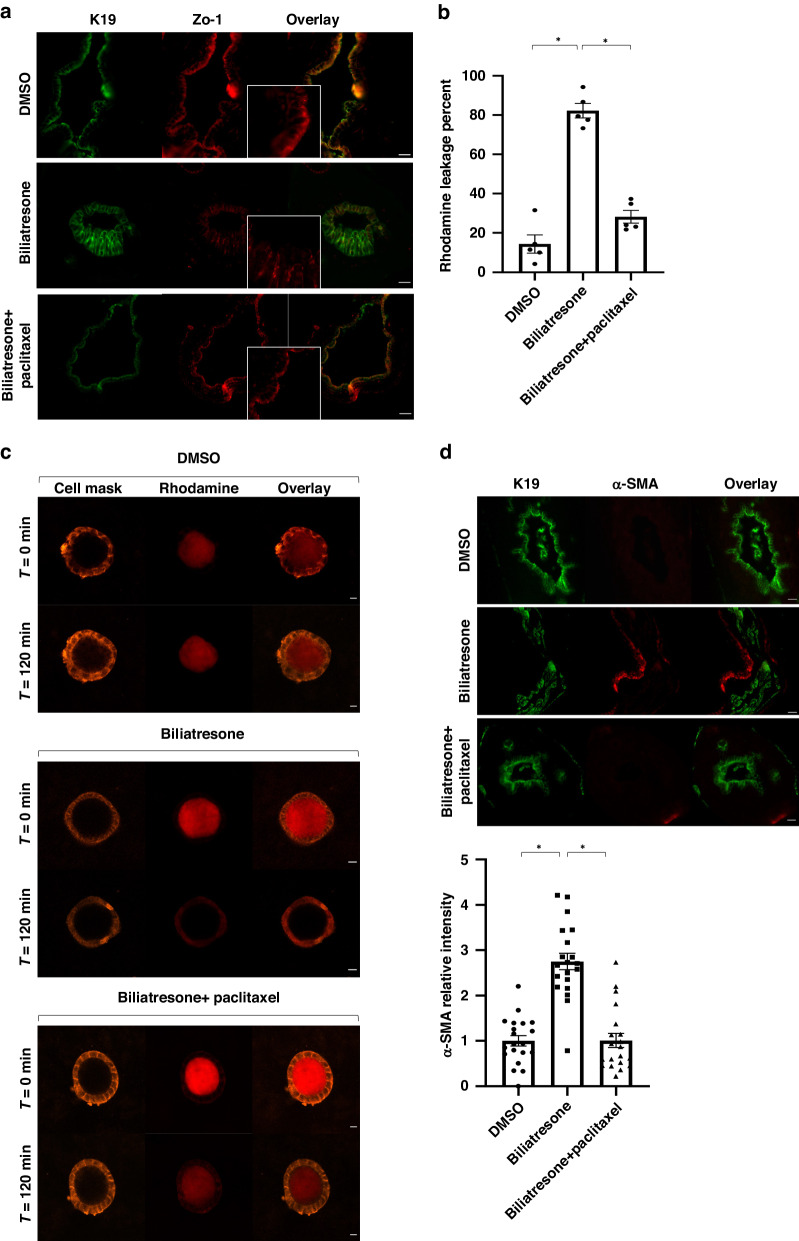
Biliatresone-induced polarity changes, increased permeability and periductal fibrosis are prevented by paclitaxel treatment. **a** Neonatal mouse EHBD were treated with biliatresone, biliatresone with paclitaxel or DMSO and immunostained for DAPI (blue) and ZO-1 (red). Scale bars: 20 µm. **b** Quantification of Rhodamine fluorescence intensity reduction. The average percent of Rhodamine fluorescence intensity reduction is shown in graph. Data are presented as the mean ± standard error, *n* = 5. (*) represents *p* < 0.01 (DMSO vs. Biliatresone *p* < 0.00001, Biliatresone vs. Biliatresone+paclitaxel *p* < 0.00001). **c** Primary Neonatal mouse cholangiocyte spheroids were loaded with Rhodamine123 and treated with the above conditions. Live cells were imaged every 20 min for 2 h after treatment, *n* = 4 (15 spheroids were evaluated). Scale bars: 20 µm. **d** Neonatal mouse EHBD were treated with the above conditions and immunostained for K19 (green) and α-SMA (red), *n* = 3, quantification of relative mean fluorescence intensity is shown in the graph below (20 images were evaluated for all groups). Error bars refer to standard errors. (*) represent *p* < 0.00001. Scale bars: 20 µm.

Lumen obstruction and changes in F-actin in spheroids are associated with mis-localization of the apical marker ZO-1^2^. These changes occur as early as 6–12 h post biliatresone treatment. We treated neonatal EHBD explants for 12 h with or without paclitaxel. Biliatresone-treated EHBD had mis-localized ZO-1 staining compared to DMSO, and the addition of paclitaxel to biliatresone prevented these changes in polarity and maintained an intact cholangiocyte monolayer (Fig. 2a).

In order to determine whether microtubule stabilization, which prevents loss of polarity, would also prevent increased epithelial permeability, we performed rhodamine 123 efflux studies in cholangiocyte spheroids. We preloaded spheroids with rhodamine 123, which accumulates in the lumen, and then treated with either DMSO or biliatresone with or without paclitaxel. Spheroids treated with DMSO maintained the majority of preloaded rhodamine 123 in the lumen (an average reduction of 14.3%) while spheroids treated with biliatresone showed rapid leakage of rhodamine 123 within 2 h (an average reduction of 82.2%, *p* < 0.00001). Paclitaxel treatment prevented most of biliatresone-induced leakage and rhodamine 123 remained inside the lumen at 2 h (an average reduction of 28.1% *p* = 0.04 compared to DMSO and *p* < 0.00001 compared to biliatresone) (Fig. 2b, c).

We wanted to assess whether microtubule stabilization would also prevent myofibroblast differentiation (increased α-SMA) in the periductal area. Biliatresone-treated EHBD resulted in increased thickness of α-SMA stain around the duct while the addition of paclitaxel to biliatresone prevented the increase in α-SMA stain and was similar to DMSO (Fig. 2d). We also treated EHBD explants with BSO and the addition of paclitaxel prevented BSO effect (Supplementary Fig. 1b).

### The effects of biliatresone are fully reversed after 24 h of washout

**Fig. 3:**
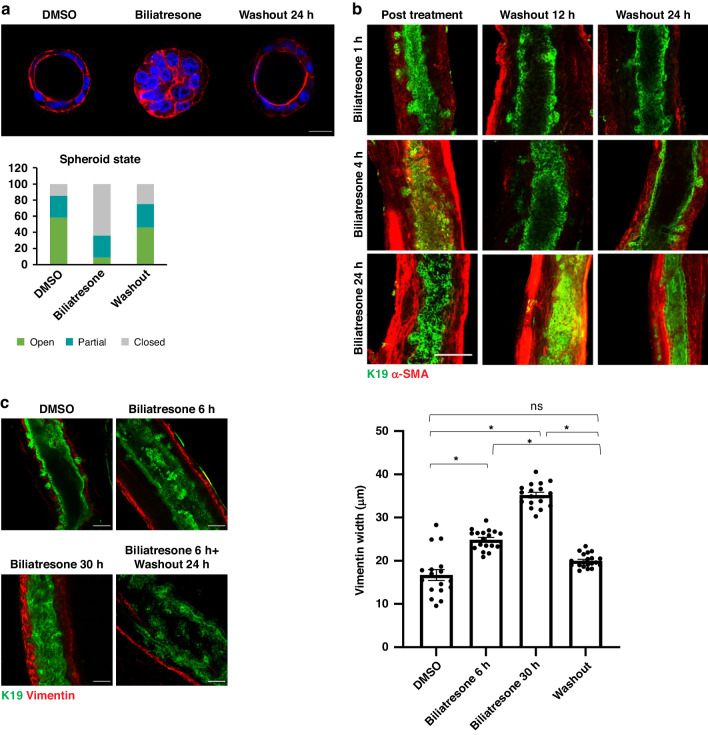
The effects of biliatresone are fully reversed after 24 h washout. **a** Primary neonatal mouse cholangiocyte spheroids were treated with biliatresone, biliatresone followed by washout or DMSO. Spheroids were stained for F-actin (red) and DAPI (blue). Spheroid state was assessed, and quantified for each condition as previously described, *n* = 3, total spheroid numbers: DMSO *n* = 82, Biliatresone *n* = 125, biliatresone followed by washout *n* = 48 Scale bars: 50 µm. **b** Neonatal EHBD were treated with biliatresone for 1, 4 and 24 h followed by washout for 12 or 24 h. Ducts were immunostained for K19 (green) and α-SMA (red), *n* = 3. Scale bars:100 µm. **c** Three-week old mouse EHBD were treated with: biliatresone for 6 or 30 h, biliatresone for 6 h followed by washout or DMSO and immunostained for vimentin (red) and K19 (green). Average vimentin thickness surrounding the lumen was quantified *n* = 3 (total of 24 ducts), (*) represents *p* < 0.01.

Next, we aimed to focus on the recovery process in spheroids after biliatresone-induced injury^2^. We treated spheroids with biliatresone, which resulted in lumen obstruction, and then washed out the biliatresone-containing media. Biliatresone washout resulted in restoration of lumens after 24 h (Fig. 3a). Biliatresone washout resulted in decrease of obstructed spheroids from 64% to 25% (*p* = 0.028); DMSO treated spheroids had 14.63% obstructed spheroids (*p* = 0.00001 compared to biliatresone).

In order to descriptively determine the effect of biliatresone over time, we treated neonatal EHBD for various time periods (1–24 h) followed by various washout times (Fig. 3b). The effect of biliatresone was time dependent, and prolonged treatment resulted in more severe obstruction and increased thickness of α-SMA stain in the peri-ductal area (Fig. 3b). A longer washout of 24 h (compared to 3 h) resulted in a better regeneration of duct tissue, with reduction of α-SMA accumulation, and a visible open lumen.

Mice EHBDs treated with biliatresone showed increased vimentin staining of the sub-epithelial layer (Fig. 3c). The vimentin layer was markedly thicker after 30 h of biliatresone treatment compared to 6 h. A 24 h washout resulted in restoration of the occluded lumen and reduction of vimentin expression (Fig. 3c).

### Molecular mechanisms underlying extrahepatic bile duct injury and recovery

**Fig. 4:**
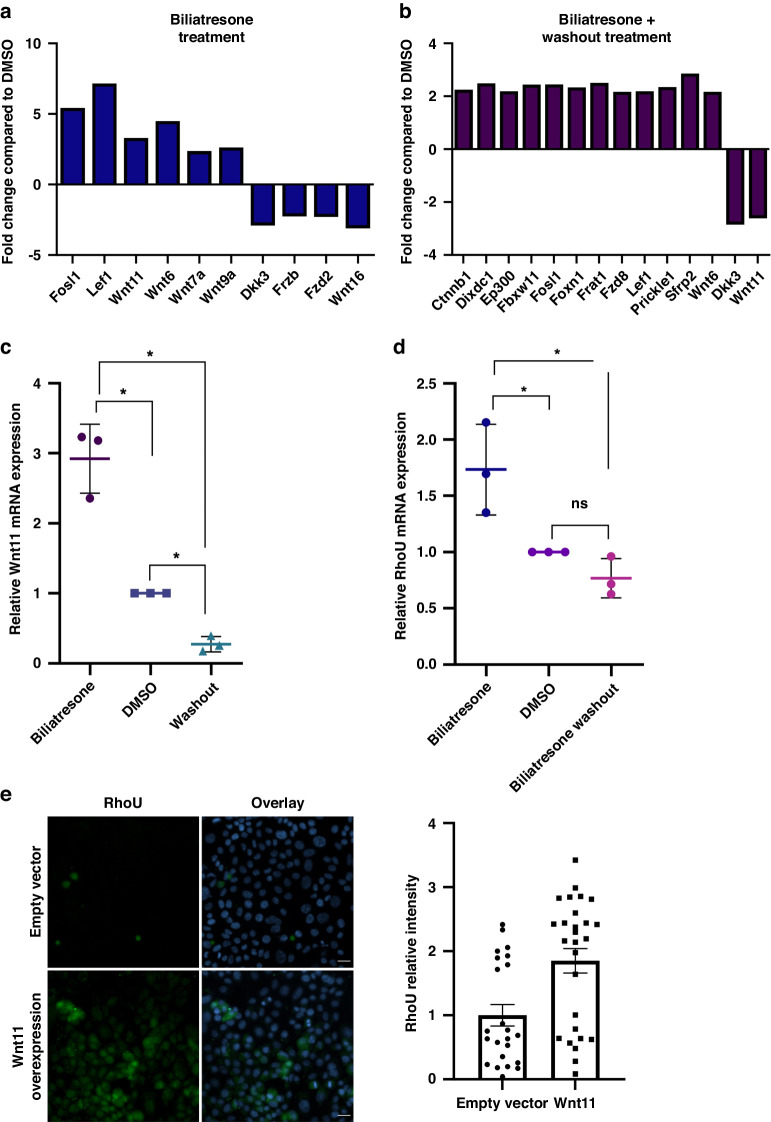
Wnt signaling pathway genes are involved in biliatresone-induced cholangiocyte injury and recovery. Wnt signaling microarray results showing gene expression changes between primary neonatal mouse cholangiocytes treated with (**a**) biliatresone and DMSO and (**b**) biliatresone washout compared to DMSO. Only genes with changes above 2 fold are shown. **c** RT-PCR of Wnt11 mRNA expression in cholangiocytes treated with biliatresone, biliatresone followed by washout or control (DMSO). Data are presented as the mean ± standard deviation, *n* = 3. (*) represents *p* < 0.05 (DMSO vs. biliatresone *p* = 0.0106, biliatresone vs. washout *p* = 0.0081, and DMSO vs. washout *p* = 0.0037). **d** RhoU mRNA expression in the same conditions as above, *n* = 3. (*) represents *p* < 0.05 (DMSO vs. Biliatresone *p* = 0.0438, Biliatresone vs. washout *p* = 0.0095 DMSO vs. washout *p* = 0.0735). **e** Cholangiocytes were transfected with an empty vector or with Wnt11 expressing plasmid. A 48 h, cells were immunostained for DAPI (blue) and RhoU (green), *n* > = 3 (23 and 27 images were quantified for empty vector and Wnt11 overexpression respectively), average values of relative mean fluorescent intensity are shown in graphs. Error bars refer to standard errors. (*) represents *p* = 0.00017. Scale bars: 20 µm.

As recovery from biliatresone-induced injury is feasible in both spheroid and explant systems, we aimed to determine the molecular mechanisms of bile duct injury and recovery and determine if those correspond. Based on our previous work^4^ we chose to further investigate the involvement of the Wnt signaling pathway genes. We used a Wnt signaling-focused PCR profiling array under three conditions: biliatresone treatment for 6 h (injury), DMSO treatment for 6 h (control) and biliatresone treatment for 6 h, followed by a 24 h washout (recovery). We set the threshold to ≥2-fold change and detected several genes that were significantly differentially expressed during cholangiocyte injury and recovery (Fig. 4a, b). Genes in the Wnt pathway that were found to be differentially expressed during both cholangiocyte injury and recovery are presented in a KEGG pathway chart (Supplementary Fig. 2 and 3). We studied selected genes based on their known physiologic role in order to determine their potential relevance in biliatresone-induced process.

Wnt11 encodes a secreted protein that acts as a ligand for members of the frizzled family of transmembrane receptors. It was also shown to possess transcription regulatory activities and promote differentiation and survival of epithelial cells^19,20^. We performed RT-PCR for Wnt11, its expression increased after biliatresone injury by 2.92 fold (*p* = 0.01) and was decreased during the recovery by 10.69 fold (*p* = 0.008) compared to injury and down by 3.66 fold compared to DMSO (*p* = 0.0037) (Fig. 4c).

Next, we wanted to examine the role of RhoU in recovery. RhoU has role in cell morphology and actin dynamics and is involved in promoting focal adhesion turnover. RhoU expression increased by 1.73 fold during injury (*p* = 0.04) and its expression decreased by 2.257 fold (*p* = 0.0095) during recovery back to baseline levels (*p* = 0.073; washout compared to DMSO) (Fig. 4d). In order to examine whether Wnt11 has an upstream effect on RhoU, we overexpressed Wnt11 in cholangiocytes using a Wnt11 plasmid. Wnt11 overexpression resulted in upregulation of RhoU (Fig. 4e and Supplementary Fig. 1c), implying that Wnt11 is upstream to RhoU.

**Fig. 5:**
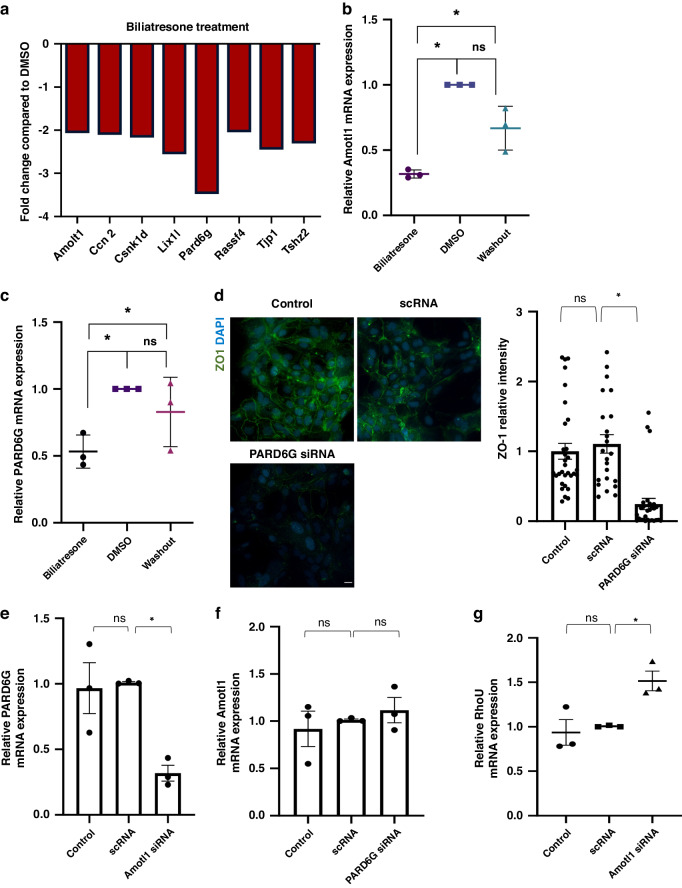
Hippo signaling pathway genes are involved in biliatresone-induced cholangiocyte injury and recovery. **a** Hippo signaling microarray results showing gene expression changes between primary neonatal mouse cholangiocytes treated with biliatresone compared to DMSO. Only genes with changes above 2 fold are shown. **b** Amotl1 mRNA expression in primary neonatal mouse cholangiocytes with biliatresone for 6 h, biliatresone for 6 h followed by fresh media washout for 24 h or control (DMSO). Data are presented as the mean ± standard deviation, *n* = 3. (*) represents *p* < 0.05 (DMSO vs. biliatresone *p* = 0.0115, biliatresone vs. washout 0.0452 DMSO vs. washout 0.1856). **c** PARD6G mRNA expression in cholangiocytes treated with biliatresone, DMSO or biliatresone for followed by washout. Data are presented as the mean ± standard deviation, *n* = 3. (*) represents *p* < 0.05 (DMSO vs. Biliatresone *p* = 0.0007, Biliatresone vs. washout 0.0369 DMSO vs. washout 0.0756). **d** Cholangiocytes were transfected with PARD6G siRNA, scRNA or control (no siRNA) and immunostained for DAPI (blue) and ZO-1 (green), *n* = 3 (31, 23 and 28 images were quantified for control, scRNA and PARD6G siRNA respectively), quantification of relative mean fluorescent intensity are shown in graph. Error bars refer to standard errors. (*) represents *p* < 0.05 (Control vs. scRNA *p* = 0.02, scRNA vs. PARD6G siRNA *p* < 0.00001). Scale bars: 20 µm. RT-PCR of cholangiocytes transfected with (**e**, **g**) Amotl1 siRNA, (**f**) PARG6G siRNA, scRNA or was used as control. Data are presented as the mean ± standard error, *n* = 3. (*) represents *p* < 0.01. **f** Control vs. scRNA *p* = 0.417, scRNA vs. Amotl1 siRNA *P* = 0.000175). **g** Control vs. scRNA *P* = 0.321, scRNA vs. PARD6G siRNA *p* = 0.005.

The Hippo signaling pathway is an evolutionarily-conserved kinase cascade pathway that regulates bile duct differentiation and homeostasis in the liver and has a role in determining biliary cell fate^21^. The Hippo signaling pathway was recently shown to regulate the Notch signaling pathway in intrahepatic cholangiocytes^21^, however it is not known if this is the same in the extrahepatic cholangiocytes. Moreover, other studies suggested that Hippo signaling pathway can regulate Wnt signaling pathway^22,23^. We used a Hippo signaling pathway-focused PCR array to detect expression in primary extra-hepatic neonatal cholangiocytes as above. Genes that were found to be differentially expressed during both cholangiocyte injury and recovery in the Hippo pathway are presented in a KEGG pathway chart (Supplementary Fig. 5 and 6). Interestingly all Hippo genes that were altered during biliatresone treatment returned to baseline during recovery (Supplementary Fig. 4, Fig. 5a).

The protein encoded by Amotl1 belongs to the Motin family of proteins, which are involved in the regulation of cell-cell junctions^24^. It is a component of tight junctions that regulates paracellular permeability and tight junction formation in vitro^25^ and is implicated in many cellular processes including the regulation of the hippo signaling pathway^26^. By RT-PCR, Amotl1 expression was reduced by 1.88 fold in biliatresone-treated cholangiocytes compared to DMSO (*p* = 0.011) and up-regulated back during recovery to an expression level that is not significantly different from DMSO (washout compared to biliatresone, *p* = 0.04; washout compared to DMSO *p* = 0.185) (Fig. 5b).

PARD6G encodes for a cell-polarity and cytoskeletal arrangement regulator protein^27^. PARD6G was downregulated after biliatresone treatment by 3.225 fold compared to DMSO (*p* = 0.0007) and upregulated during recovery by 2.13 fold to levels similar to basal levels (washout compared to biliatresone, *p* = 0.03; washout compared to DMSO, *p* = 0.075) (Fig. 5c). Interestingly, silencing PARD6G resulted in a decreased expression of ZO-1 in primary cholangiocytes, indicating the impaired polarity of the cells and consequently the role of PARD6G in maintaining the intact cholangiocyte polarization state (Fig. 5d, Supplementary Fig. 4b). Next, we silenced Amotl1, which resulted in a significant downregulation of PARD6G, but not vice versa (Fig. 5e, f). This implies that Amotl1 is upstream of PARD6G. Finally, silencing Amotl1 led to upregulation of RhoU, but did not affect Wnt11 (Fig. 5g). Suggesting that Hippo pathway genes may have some upstream regulatory effect on Wnt signaling pathways, but this process is complex and not all Wnt genes are downstream to Hippo.

## Discussion

Biliary atresia is a cholangiopathy of early infancy with severe outcomes that presents with EHBD obstruction and disruption of bile flow^1^. Understanding the molecular mechanisms of cholangiocyte injury and recovery are of immense importance.

**Fig. 6:**
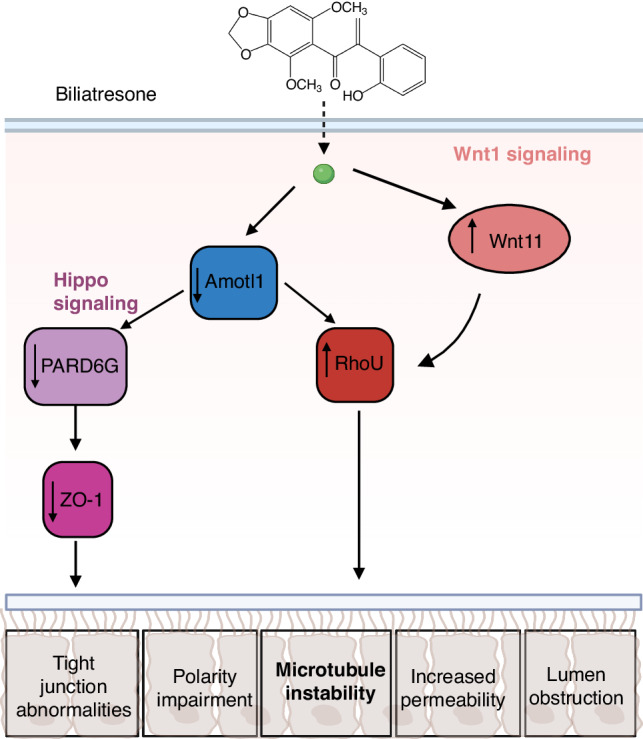
Model of proposed pathway of biliatresone-induced injury. Biliatresone causes decreased levels of Amotl1, a Hippo signaling pathway member, which lead to downregulation of PARD6G which in turn causes the downregulation of Zo-1, which results in the disruption of tight junctions. Amotl1 downregulation and Wnt11 upregulation were both shown to lead to the upregulation of RhoU/Wrch1, ultimately leading to polarity impairment, tight junction abnormalities, increased permeability and lumen obstruction. This Figure was created with BioRender.com.

Here we showed that microtubule instability plays a key role in cholangiocyte injury. Microtubule stabilization prevents cholangiocyte injury and maintains lumen integrity in biliatresone-induced BA. Microtubule stabilization not only prevents cholangiocyte injury and the increased permeability induced by biliatresone, but also peri-ductal myofibroblast accumulation. Wnt and Hippo signaling pathways play a role in biliatresone-induced injury and recovery processes (Fig. 6). RhoU and Wnt11 are upregulated following biliatresone injury and downregulated during recovery and that Wnt11 is upstream of RhoU expression. In the Hippo signaling pathway, most genes that were upregulated during injury were reduced back to their basal level during recovery. Amotl1 is upstream to PARD6G and decreased expression of PARD6G resulted in ZO-1 reduction and subsequently impaired cell polarity.

Microtubule stability plays a crucial role in the structure and function of epithelial cells. For instance, an integrin-ILK-microtubule network orients cell polarity and lumen formation in glandular epithelium^28^. Microtubules are also essential for the formation and function of primary cilia, which activate intracellular signaling pathways upon bile flow^28–30^. Primary cilia was shown to be relevant in human BA, rotavirus BA murine model and biliatresone-induced cholangiocyte injury^3,31,32^.

Biliatresone-induced increased epithelial permeability is prevented by microtubule stabilization. Interestingly not only cholangiocyte damage was prevented, but also myofibroblast staining in the periductal area. We previously showed that glycochenodeoxycholic acid (GCDCA), when administered to the basolateral layer of mice EHBD, caused peri-ductal fibrosis^33^. Increased permeability and bile leak may propagate fibrosis. Increased periductal vimentin expression induced by biliatresone goes along with other cholangiopathies in the Mdr2 knockout mouse model as well as human PSC patients^34^, and also reported in BA patients^35^. We showed here the potential for the reversibility of this process, perhaps in early stages of injury.

Studying bile duct recovery mechanisms is of extreme importance. RhoU which is important in biliatresone-induced injury and has a role in disruption of cell polarity^4^, goes back down to its basal levels during recovery. Wnt11 is another Wnt signaling gene that we found to be upregulated due to biliatresone-induced injury and was downregulated to more than 3.5 times below basal levels during recovery. Wnt11 protein is a GTPase activator that is involved in cell fate and differentiation^36,37^. Interestingly, Wnt11 is activated by Transforming Growth Factor (TGF)-β in renal epithelial cells and is upregulated in a mouse model of renal fibrosis^38^. Furthermore, the reduction of Wnt11 leads to a significantly thinner fibrotic capsule around silicone implants^39^; reduction of Wnt11 levels during recovery might thus explain the reduction of **α**-SMA deposition in the EHBD that was seen during recovery. Overexpression of Wnt11 resulted in the upregulation of RhoU, suggesting that both are in the same pathway of injury and that Wnt11 is upstream to RhoU.

The Hippo signaling pathway is a key regulator of cell fate, that is activated in cholangiocytes during cholestatic diseases in human^40^. Recent studies showed that it may regulate the Notch signaling pathway in intrahepatic cholangiocytes^21^ and the Wnt signaling pathway in other systems^23^, we were thus interested to examine its role in biliatresone-induced injury and recovery. Amotl1 and PARD6G are Hippo signaling pathway genes that were downregulated in biliatresone-induced injury and both were upregulated during recovery. Amotl1 was upstream to PARD6G. The protein encoded by Amotl1 gene is a peripheral membrane protein that is a component of tight junctions, acting to control paracellular permeability and maintain cell polarity^41^. Interestingly, Amotl1 was found to facilitate proliferation of biliary epithelial cells^42^ thus this may indeed play a role in bile duct recovery. Amotl1 silencing results in the upregulation of RhoU. This suggests a complex interplay between Hippo and Wnt pathways. Others showed Amotl1’s role in inhibition of the Wnt/beta-catenin signaling pathway^43^. PARD6G encodes for an adapter protein involved in asymmetrical cell division and cell polarization processes^44^. In our system, the downregulation of PARD6G by biliatresone resulted in reduced ZO-1 expression, indicating tight junction abnormalities and polarity impairment.

Collectively, biliatresone-induced injury is prevented by microtubule stabilization and may be reversed after injury. Both the Hippo and Wnt signaling pathways are involved in these processes and there is an interplay between the pathways. There are currently no directed therapies for biliary atresia. Further human studies exploring the mechanisms of recovery are needed to potentially identify future therapeutics.

## Supplementary information

Supplementary Information

## Data Availability

All data generated or analyzed during this study are included in this published article [and its supplementary information files].
