# Supplementary material for: Biliary atresia: insights into mechanisms using a toxic model of the disease including Wnt and Hippo signaling pathways and microtubules
Source: Pediatr Res. 2024 Jun 25;97(1):184–94. doi: 10.1038/s41390-024-03335-9 (PMC11798875; doi:10.1038/s41390-024-03335-9)
Supplement: Supplementary file 1 — Supplementary Information [file 41390_2024_3335_MOESM1_ESM.pdf]

Supplementary Figure 1

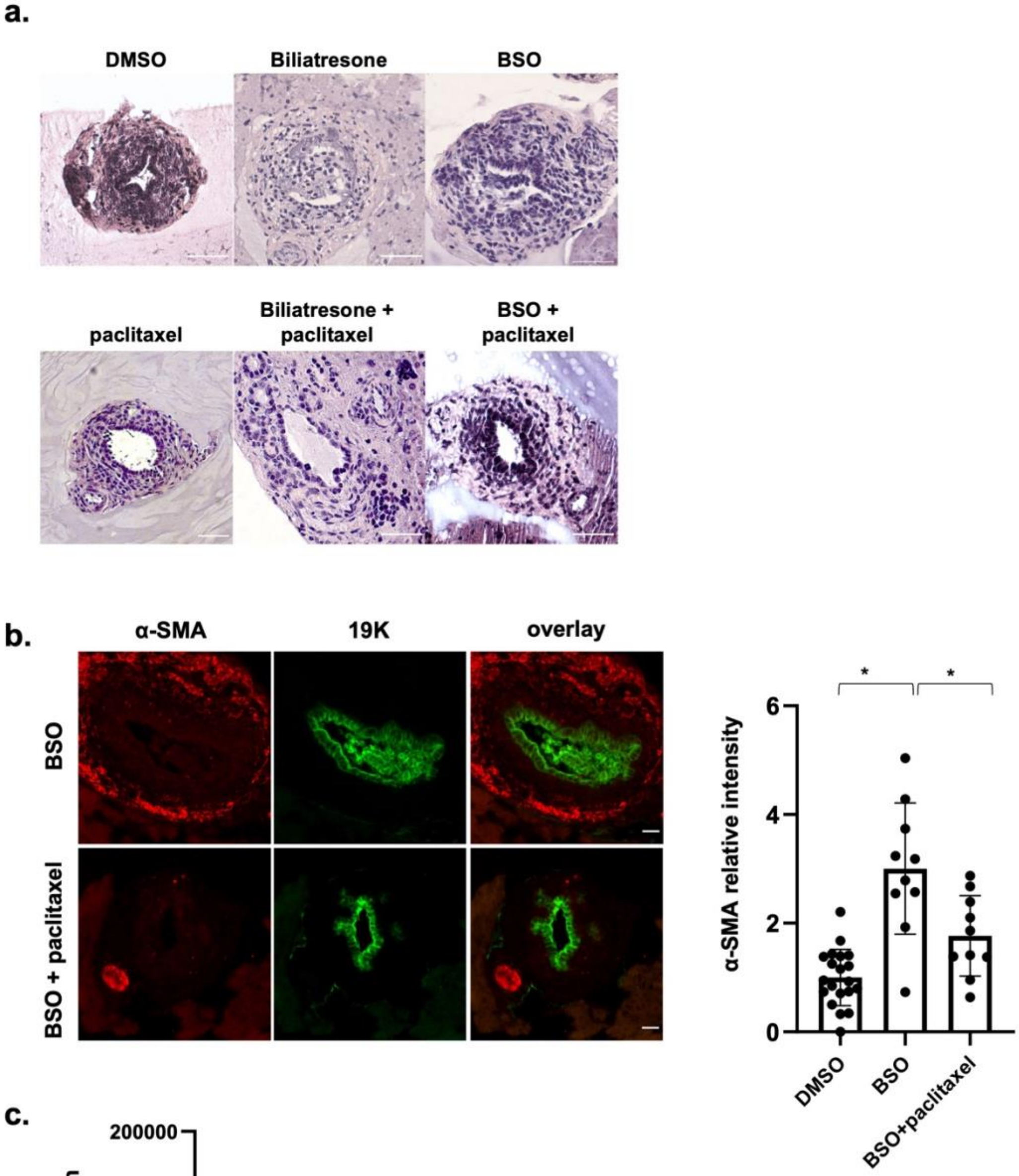

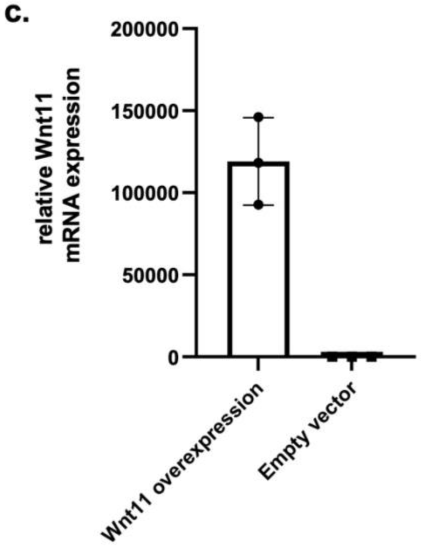

Supplementary Figure 1. The effects of biliatresone are mimicked by BSO and nocodazole and prevented by paclitaxel. (A) EHBD were dissected and incubated for 6h with biliatresone, BSO, biliatresone+paclitaxel, BSO+paclitaxel, paclitaxel or DMSO as control. The ducts were then sectioned crosswise and stained for hematoxylin and eosin. Scale bars: 50 µm. (B) Neonatal mouse EHBD incubated for 6h in a high-oxygen environment, treated with BSO or BSO+paclitaxel and immunostained for K19 (green) and α-SMA (red), n=3. α-SMA staining quantification (relative mean fluorescence intensity) shown in graph. Standard error reflected as error bars, (*) represents p<0.01 (DMSO vs. BSO p=0.0004, BSO vs. BSO+paclitaxel p=0.014). Scale bars: 20 µm. (C) Wnt11 relative mRNA expression. Wnt11 messenger RNA expression in primary neonatal (3 days old) cholangiocytes, transfected with an Empty vector as control or with Wnt11 expression plasmid and incubated for 48h. Data are presented as the mean ± standard errors, n=3. (*) represents p < 0.01 (p=0.016).

Supplementary Figure 2

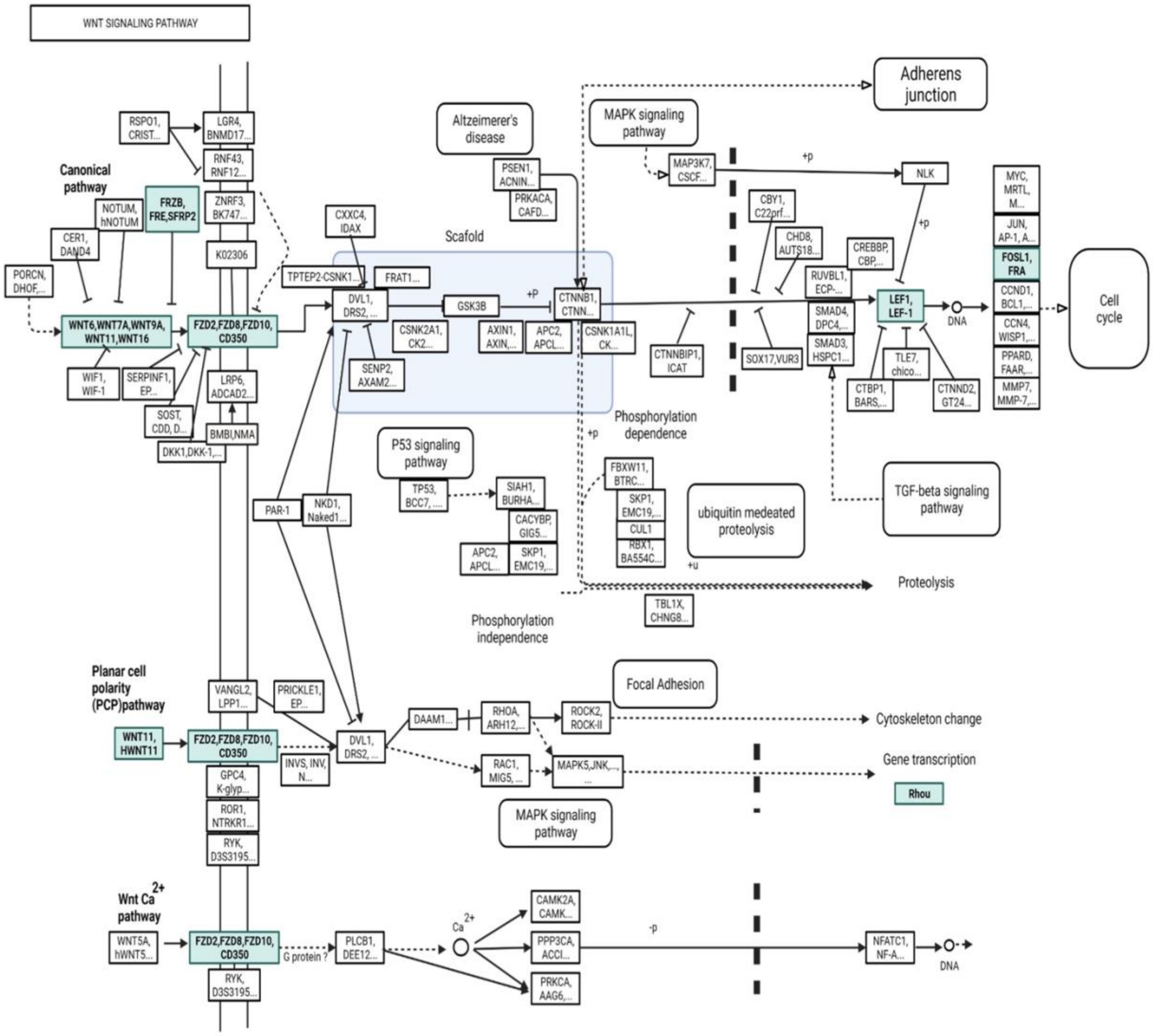

Supplementary Figure 2. Wnt signaling pathway involvement in biliatresone-induced cholangiocyte injury. Wnt signaling pathway genes that were shown to change significantly following Biliatresone treatment compared to DMSO is presented in a KEGG pathway scheme. Highlighted genes are affected in the process.

Supplementary Figure 3

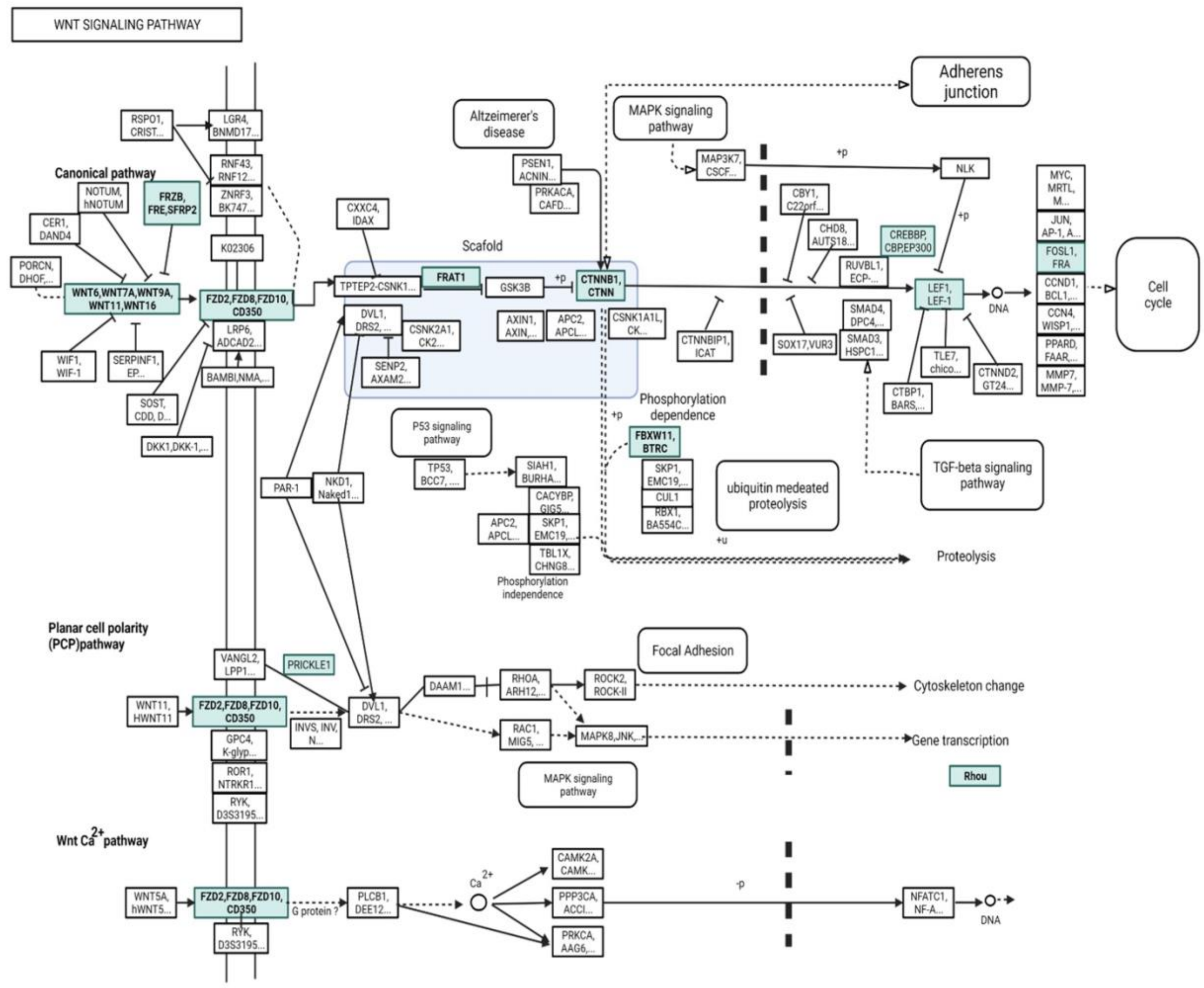

Supplementary Figure 3. **Wnt signaling pathway involvement in the recovery from biliatresone-induced cholangiocyte injury.** Wnt signaling pathway genes that were shown to change significantly as a result of biliatresone treatment, followed by a washout compared to DMSO are presented in a KEGG pathway scheme. Highlighted genes are affected in the process.

Supplementary Figure 4

a.

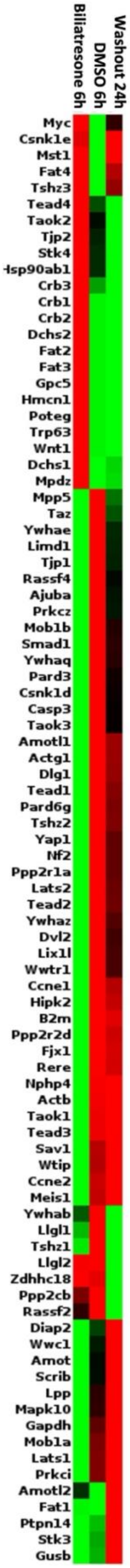

b.

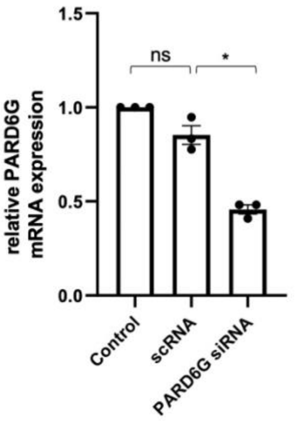

Supplementary Figure 4. Hippo signaling microarray heat map and PARD6G relative mRNA expression. (A) Hippo signaling array heat map for primary cholangiocytes treated with (1) Biliatresone for 6 hours (injury), (2) DMSO for 6 hours (control) and (3) Biliatresone 6 hours followed by 24 hours of washout (recovery). (B) Primary neonatal cholangiocytes were transfected with PARD6G siRNA, scRNAor control (no siRNA). After 48h cells were lysed and mRNA expression was assessed by RT-PCR. Data are presented as the mean ± standard error, n=3. (*) represents $p < 0.01$. (Control vs. ScRNA P=0.09, ScRNA vs. PARD6G siRNA P=0.006).

Supplementary Figure 5

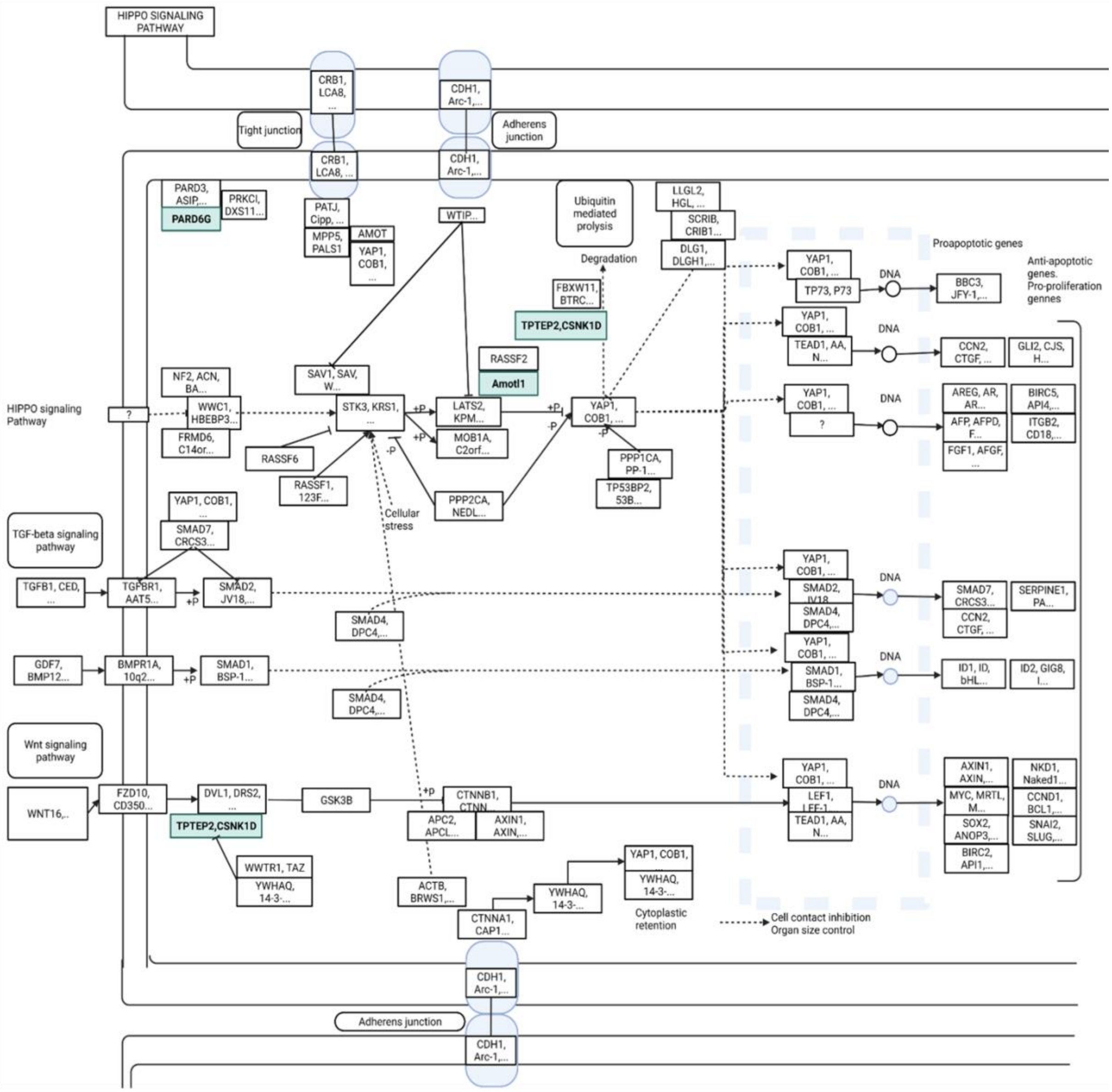

Supplementary Figure 5. Hippo signaling pathway involvement in biliatresone-induced cholangiocyte injury. Hippo signaling pathway genes that were shown to change significantly following biliatresone treatment DMSO are presented in a KEGG pathway scheme. Highlighted genes are affected in the process.

Supplementary Figure 6

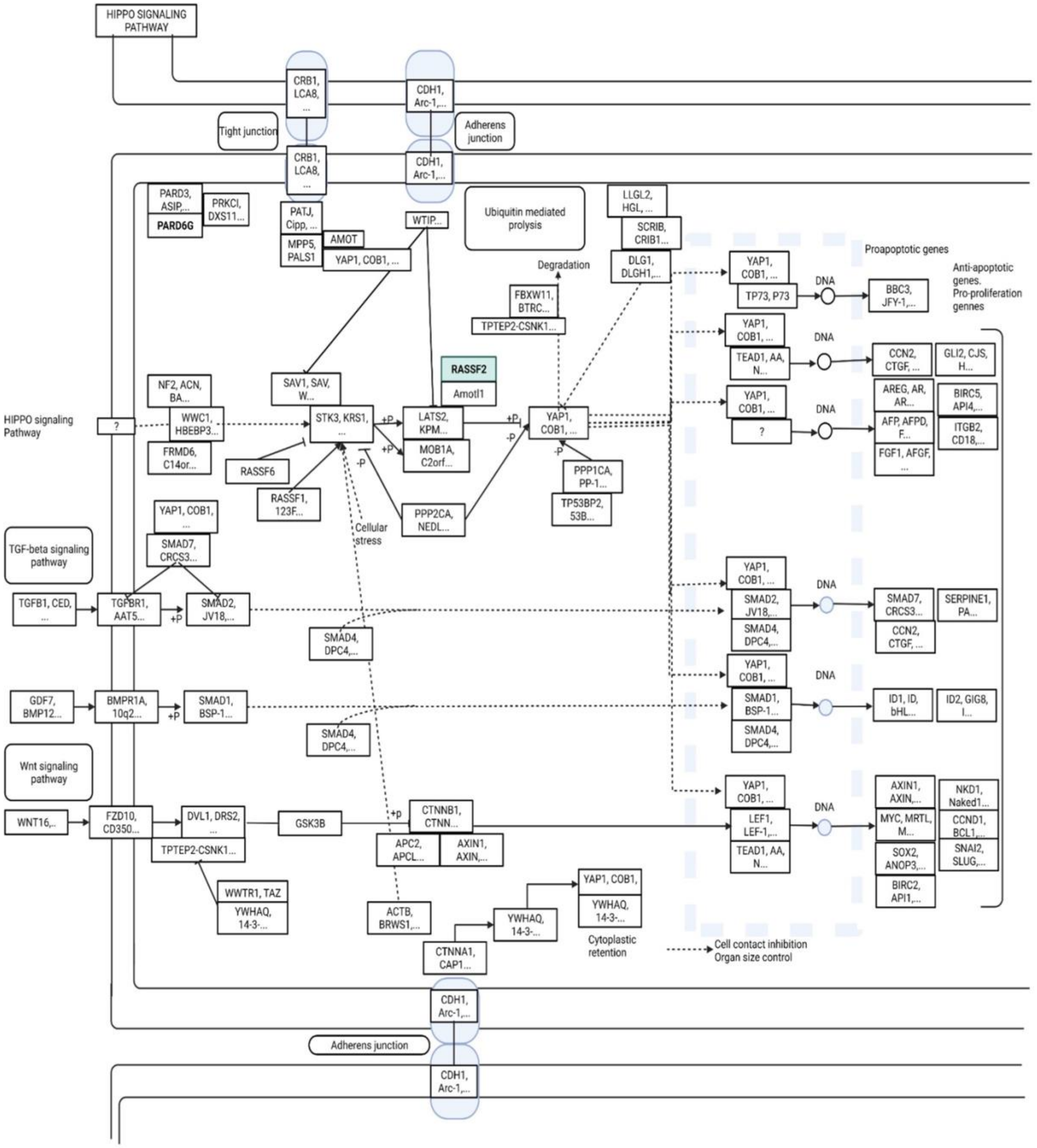

Supplementary Figure 6. Hippo signaling pathway involvement in the recovery from biliatresone-induced cholangiocyte injury. Hippo signaling pathway genes that were shown to change significantly as a result of biliatresone treatment, followed by a washout, are compared to DMSO and presented in a KEGG pathway scheme. Highlighted genes are effected in the process.
